# Understanding the neurological mechanism involved in enhanced memory recall task following binaural beat: a pilot study

**DOI:** 10.1007/s00221-021-06132-6

**Published:** 2021-07-07

**Authors:** Muhammad Danish Mujib, Muhammad Abul Hasan, Saad Ahmed Qazi, Aleksandra Vuckovic

**Affiliations:** 1 Department of Biomedical Engineering, NED University of Engineering and Technology, Karachi, Pakistan; 2 Department of Biomedical Engineering, Faculty of Engineering Science and Technology, Hamdard University, Karachi, Pakistan; 3 Neurocomputation Lab, National Center of Artificial Intelligence, Karachi, Pakistan; 4 Department of Electrical Engineering, NED University of Engineering and Technology, Karachi, Pakistan; 5 Biomedical Engineering Division, University of Glasgow, Glasgow, G12 8QQ UK

**Keywords:** Binaural beat, EEG power, Cognitive score, Reaction time, Memory, Imaginary coherence

## Abstract

Binaural beat (BB) is a promising technique for memory improvement in elderly or people with neurological conditions. However, the related modulation of cortical networks followed by behavioral changes has not been investigated. The objective of this study is to establish a relationship between BB oscillatory brain activity evoked by stimulation and a behavioral response in a short term memory task. Three Groups A, B, and C of 20 participants each received alpha (10 Hz), beta (14 Hz), and gamma (30 Hz) BB, respectively, for 15 min. Their EEG was recorded in pre, during, and post BB states. Participants performed a digit span test before and after a BB session. A significant increase in the cognitive score was found only for Group A while a significant decrease in reaction time was noted for Groups A and C. Group A had a significant decrease of theta and increase of alpha power, and a significant increase of theta and decrease of gamma imaginary coherence (ICH) post BB. Group C had a significant increase in theta and gamma power accompanied by the increase of theta and gamma ICH post BB. The effectiveness of BB depends on the frequency of stimulation. A putative neural mechanism involves an increase in theta ICH in parieto-frontal and interhemispheric frontal networks.

**Supplementary Information:**

The online version contains supplementary material available at 10.1007/s00221-021-06132-6.

## Introduction

Binaural beat (BB) occur when two sinusoidal waves at slightly different frequencies are presented separately to each ear and the brain perceives a sound with a frequency corresponding to the difference of these two frequencies. For example, tones of 400 Hz and 410 Hz presented to the right and left ears, respectively, produce a BB sound of 10 Hz. Studies found that frequencies of around 400 Hz with a maximum difference of up to 35 Hz between two frequencies induces the strongest behavioral and psychological effects (Colzato et al. 2018; Garcia-Argibay et al. 2019). BB is believed to originate subcortically in the pons, in the medial nucleus of the superior olivary complex, the first nucleus in the auditory pathway to receive bilateral input (Kuwada et al. 1979; Wernick and Starr 1968). BB can entrain cortical activity at both the specific frequency of the beat (Draganova et al. 2008; Pratt et al. 2009) and cross-frequency modulations, such as theta beats driving interhemispheric alpha synchronization (Solca et al. 2016).

There is a debate about the putative mechanism of BB that has been attributed to either entrainment or interhemispheric coherence (Garcia-Argibay et al. 2019). Nevertheless, there is a growing support for the claim that binaural auditory beats affect cognition and psychophysiological states.

The effect of theta, alpha, beta, and gamma BB protocols have been studied previously in clinical populations and healthy individuals by measuring cognitive functions and analyzing electrophysiological changes (Beauchene et al. 2017; Goodin et al. 2012). Clinical symptoms targeted with different BB protocols include depression, memory, parasympathetic activation, and self-reported relaxation (McConnell et al. 2014), heart rate variability (Palaniappan et al. 2015), anxiety (Chaieb et al. 2015; Padmanabhan et al. 2005), hypertension, sleep, pain, schizophrenia, Alzheimer's disease, and mental states (Garcia-Argibay et al. 2019; Gkolias et al. 2020; McDermott et al. 2018; Wahbeh et al. 2007). Studies also reported improvement of vigilance (Lane et al. 1998), increase in hypnotic susceptibility (Brady and Stevens 2000), or increase in some forms of creativity (Reedijk et al. 2013). Several studies also reported behavioral changes for clinical symptoms targeted with BB (Carter 2008; Kennel et al. 2010; Kennerly 2004). However, some other studies failed to find an improvement in behavioral or physiological outcomes such as attention (Crespo et al. 2013), blood pressure or heart rate variability (Carter 2008), memory (Wahbeh et al. 2007), anxiety (Le Scouranec et al. 2001), and reduction of symptoms in children diagnosed with attention deficit hyperactivity disorder (Kennel et al. 2010).

The effect of different types of BB on memory functions has been studied in healthy populations including the elderly, as well as in patients with neurological conditions that affect memory, such as Alzheimer’s disease, traumatic brain injury, Parkinson’s disease, and pain (Calomeni et al. 2017; McDermott et al. 2018; McMurray 2006). A study conducted on an elderly population with Alzheimer’s and Parkinson’s disease found enhanced memory functions accompanied with the enhanced alpha activity (Calomeni et al. 2017; McMurray 2006). Studies additionally reported enhanced memory performance with BB in patients with Parkinson’s disease (Gálvez et al. 2018). A meta-analysis (Garcia-Argibay et al. 2019) concluded that theta, alpha, beta, and gamma BB may influence performance in memory task, with alpha, beta, and gamma BB being positively related to the performance, while results of theta BB were inconclusive.

Notwithstanding improvements in clinical symptoms and behavioral outcomes, most of the published studies lack a detailed analysis on brain entrainment following BB stimulations. Most frequently reported outcomes are EEG spectral changes in different frequency bands, notably highest for the gamma band at 40 Hz, over the frontal and parietal regions (Draganova et al. 2008; Pastor et al. 2002; Schwarz and Taylor 2005). Other BB frequencies that resulted in an increase of EEG power in the corresponding frequency bands included the theta band power over the frontal, parietal, and temporal regions (Draganova et al. 2008; Kennerly 2004; Pastor et al. 2002; Pratt et al. 2009; Schwarz and Taylor 2005) and the alpha and beta band power (Fitzpatrick et al. 2009; Frederick et al. 1999; Gao et al. 2014; Solca et al. 2016). However, some studies reported no changes following BB stimulation (Gooding et al. 2012; Crespo et al. 2013; Castro San Cristóbal 2019).

Recently (Perez et al. 2020) performed EEG analysis of cortical and subcortical structures responses to BB in theta and gamma range and related changes in mood. They showed that both beat types entrained the brain at their beat frequencies, with monaural conditions eliciting the highest response when compared to binaural conditions. They analyzed phase locking values and imaginary coherence during both conditions but failed to show any effect of BB on mood change.

On the other hand, while several studies employed BB stimulation for improvement of cognitive functions such as memory and attention, the related modulation of cortical networks was not investigated (Kennerly 1994; Kraus and Porubanová 2015). Reporting changes in neurological measures such as EEG power spectrum and effective connectivity among cortical regions responsible for processing memory functions alongside the changes in cognitive measures is necessary to understand the mechanism of BB stimulation.

The objectives of this study are to (1) to explore the effect of BB at different frequencies on memory recall task with different levels of difficulty (2) to establish a relation between a behavioral task and related neurological responses, and (3) to propose the putative mechanism of BB.

## Methods

### Participants

Sixty healthy participants (44 males, 16 females 25.73 ± 2.02 years old), took part in this study. The exclusion criteria were a self-reported history of substance abuse, dependence, brain surgery, tumor, or intracranial mental implantation. Participants were divided into three Groups; A, B, and C, where each group comprised 20 participants. Each group followed the same experiment protocol but with different frequency of BB. Alpha BB (10 Hz), beta BB (14 Hz), and gamma BB (30 Hz) were provided for Groups A, B, and C, respectively. All participants signed the informed consent. The study was approved by the University Ethical and Advanced Studies Research Committee.

### Experimental procedures

**Fig. 1:**
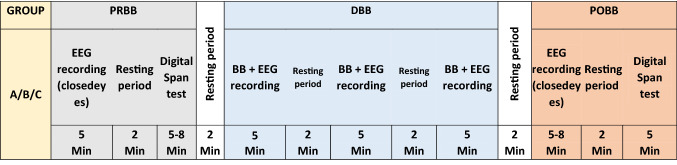
Experiment protocol for binaural beat stimulation for three groups (Group A: alpha BB, Group B: Beta BB, Group C: Gamma BB). PRBB represents pre BB, DBB represents during BB, and POBB represents post BB

The experimental procedures are shown in Fig. 1. Participants were asked to sit in a comfortable position and relax. EEG was recorded in a relaxed eye closed state for 5 min. This was followed by a cognitive memory test, i.e., a digit span test, lasting 5–8 min. After completion of the cognitive test, participants received BB stimulation (all three Groups A, B, and C) over a period of 15 min, split into three sub-session, lasting 5 min each in an eyes closed state. Participants were allowed to take a few minutes rest between sub-sessions. Following completion of all three sessions of BB stimulation, EEG was recorded in eyes closed condition for 5 min. Finally participants repeated a digit span test.

#### BB stimulation

Three different BB stimulation conditions were evaluated during the course of the study: (1) 10 Hz BB (R: 410 Hz, L: 400 Hz), (2) 14 Hz BB (R: 414 Hz, L: 400 Hz), and (3) 30 Hz BB (R: 430 Hz, L: 400 Hz). The tones were presented to the right and left ears are labelled R and L, respectively. The tones were created using a software Adobe Audition v3.0 (Gao et al. 2014; Vernon et al. 2014). The stimulus volume, played through stereo headphones (MDR-NC7, Sony) (Al-Shargie et al. 2019; Beauchene et al. 2016, 2017), was set by the participants at the start of the session to a comfortably loud level. The stimuli were delivered at minimum intensities of 50 dB above threshold (Al-Shargie et al. 2019).

#### Behavioral task

The digit span test was presented as a random sequence of digits to the participants. The test was performed prior and post BB stimulation and was composed of five rounds, based on the length of a digit sequence. Here, the simplest sequence contained four digits in the first round (low level of difficulty) and the most complex consisted of eight digits in the last round. New, random sequences of digits were shown on the screen each time, for each difficulty level, for both PRBB and POBB states. There were a total of ten digit series displayed in each of five rounds. To ensure the visibility and clarity of presentation, the digits were written in Arial font, size 24 in black color on a white background. Each digit was displayed at the center of the screen for 500 ms followed by another digit. Participants were asked to memorize and recall the digits in the order of presentation and to enter them via a keyboard immediately after all the digits were displayed. Performance of the participants was evaluated with respect to the maximum number of correct digits recalled and the time required to complete each round. Participants were allowed to proceed to the next round only when if they achieved a minimum 60% score. The score in each round was calculated by dividing the number of digits sequences correctly recalled with total numbers of sequences per trial (Eq. 1). The final score was calculated by dividing the sum of scores obtained in each round with the total numbers of rounds that a participant completed.

1 $${\text{Score}}\,\;\left( {\text{\% }} \right) = \frac{{{\text{Total}}\,\;{\text{number}}\,\;{\text{of}}\;{\text{digits}}\;\,{\text{correctly}}\;\;{\text{recalled}}}}{{{\text{Total}}\;\,{\text{number}}\;\,{\text{of}}\;\,{\text{trials}}\;\;{\text{appeared}}}} \times {100}$$

### EEG recording

Brain activity was recorded using a wireless EEG device EPOC (Emotiv Technology Inc., USA) from 14 scalp locations (AF3, F7, F3, FC5, T3, P7, O1, O2, P8, T4, FC6, F4, F8, AF4) in accordance with the standard 10–20 international system (Jasper 1958). EEG was recorded in pre (PRBB), during BB stimulus (DBB), and post BB (POBB) states. The sampling frequency was set at 128 Hz. One mastoid (M1) sensor acted as a ground reference point to which the voltage of all other sensors was compared. The other mastoid (M2) was a feed-forward reference that reduced external electrical interference (Badcock et al. 2013). Electrode impedance was kept below 5kΩ. Saline liquid was used to obtain good conductivity. Data were acquired in a quiet and ventilated room.

#### EEG signal processing

##### Artefact removal

EEG data were processed using MATLAB (R2018a, Natick, MA, USA). Detrending was applied on raw EEG data to adjust the baseline by removing a DC offset. For removing 50 Hz artefact, a 5th order Butterworth band-pass IIR filter (1–45 Hz) was applied. Matlab command “butter (n, Wn, ftype)” was used to determine filter coefficients by setting filter order *n*, frequency range *Wn*, and filter type *ftype*. Finally, “filter (*b*, *a*, x)” command was used to get a required filtered output, where *a* and *b* are filter coefficients and *x* are raw EEG data as an input. EEG data with eye blinks, ocular movements and EMG artifacts were removed based on a visual inspection. The rejection rate was less than 10%.

##### Calculating EEG power

The power spectral density of EEG signals was computed using Welch’s averaged modified periodogram method implemented in Matlab; this is a non-parametric method which provides smoother frequency spectrum than the raw fast Fourier transform output. A sliding Hanning window of 4 s, with an overlap of 2 s, was applied to improve the estimation quality by controlling the spectral leakage and variance in the data. The number of FFT points was set to 512 to have a PSD estimate with a frequency resolution of 0.25 Hz (the frequency sampling rate was 128 Hz). Following this, relative EEG power was calculated by normalizing absolute power at each frequency band with the sum of absolute power between 1 and 45 Hz for all channels. The relative power was then averaged in four frequency bands, theta (4–8 Hz), alpha (8–13 Hz), beta (14–30 Hz), and gamma (30–40 Hz).

##### Analysis of imaginary coherence

The imaginary coherence is not affected by volume conduction and hence it is expected to provide a true interaction between sensors by eliminating the extraneous coherence or self-interaction. It should be noted though that ICH is unable to disentangle spurious from real immediate connectivity.

We used ICH to explore the influence of different BB stimulation frequencies on the frequency specific interaction among different brain regions. In this study, the ICH analysis was performed on EEG data recorded in PRBB, DBB, and POBB states. The ICH was calculated using a method outlined in (Nolte et al. 2004) and presents the measure of a linear relationship (i.e., correlation) between two signals (in this case, electrodes) at specific frequencies.

2 $${\text{ICH = imag}}\left( {\frac{{{\text{CPSD}}\left( {x,y} \right)}}{PxxPyy}} \right)$$

where ${I}_{\mathrm{coh}}=\mathrm{Imaginary Coherence},$ ${\mathrm{CPSD}}_{\left(x,y\right)}$= Cross power spectral density between channels *x* and *y*, ${P}_{xx}=\mathrm{Power for channel }x, {P}_{yy}=\mathrm{Power for channel y}.$

${\mathrm{CPSD}}_{\left(x,y\right)}$, ${P}_{xx}$ and ${P}_{yy}$ were computed using Welch’s averaged modified periodogram method. A sliding Hanning window of 4 s, with an overlap of 2 s, was applied. The number of FFT points was set to 512 to get a frequency resolution of 0.25 Hz (the frequency sampling rate was 128 Hz). The ICH was then calculated among all channels for each frequency bin till 45 Hz using Eq. (2). The values of ICH are both positive and negative. The positive ICH_*x*->*y*_ represents the flow of information from a channel ‘*x*’ (source) to a channel ‘*y*’ (sink) while negative ICH_*x*->*y*_ demonstrates that a channel ‘*x*’ acts as a sink. The absolute value of ICH represents the strength of connectivity while sign of ICH represents directionality (source or sink). Hence, the average of absolute values of ICH was calculated in four frequency bands: theta (4–8 Hz), alpha (8–13 Hz), beta (14–30 Hz), and gamma (30–40 Hz).

### Statistical analysis

The demographic and baseline performance data of all 60 participants which includes participants' age, cognitive score and reaction time (digit span test) in PRBB state were compared between each two groups (Group A, B, and C) using unpaired *t* test (*p* < 0.05).

The Cohens method was applied for finding the effect size and to demonstrate whether the effects of training have practical importance; ensuring that significant behavioral changes in POBB state are not due to false positives (Cohen 1988). The effect size was calculated for both changes in scores and reaction times. Mean behavioral scores of two groups were subtracted and divided with a pooled standard deviation (see Eq. (3)). Similarly, the effect size was calculated on the mean and standard deviation of reaction times between each two groups (A vs B, A vs C, and B vs C) using same Eq. (3). The values of the effect size larger than 0.8 were considered as large while values between 0.4 and 0.8, and less than 0.4 were considered as medium and low effect sizes.

For studying neurological changes, a paired *t* test was applied to compare between power recorded in PRBB state with the power recorded in DBB and POBB states.

3 $${\text{Cohen}}\;d = \frac{{\overline{X}_{1} - \overline{X}_{2} }}{{\sqrt {\frac{{{\text{SD}}_{1} \left( {n_{1} - 1} \right) + {\text{SD}}_{2} \left( {n_{2} - 1} \right)}}{{\left( {n_{1} + n_{2} - 2} \right)}}} }},$$

where $\overline{X }$ are mean values, SD_1,2_ are standard deviations, and *n*_1,2_ are sample sizes of two variables.

The impacts of all three types of BB were assessed by comparing changes in behavioral and neurological outcomes in DBB and POBB states as compared to the PRBB state. The Wilcoxon signed rank test was applied for comparing changes in memory score and total time for task completion between POBB and PRBB states while linear regression analysis was performed in both states separately to determine the association of cognition with the length of a digit sequence. For studying neurological changes, a paired *t* test was applied to compare between the relative EEG power recorded in PRBB state with the power recorded in DBB and POBB states. To compare the ICH (absolute values) measured in PRBB state with ICH values in POBB and DBB states, a paired *t* test was applied which was then corrected for multiple comparisons using false discovery rate for controlling type-I error (Benjamini and Yekutieli 2001).

The N way Analysis of Variance (ANOVAN) was performed in Matlab between the total number of sinks or sources as independent variable and groups (A, B, C), states (DBB, POBB as compared to PRBB state), cortical areas (anterior, occipital-posterior, temporal), and frequency bands (theta, alpha, beta, and gamma) as independent variables. This was followed by a *t* test between variables which showed significant differences. Holm–Bonferroni correction for multiple comparison was applied.

All behavioral and neurological features were initially tested for normal distribution using Shapiro–Wilk test before doing parametric statistical analysis in the whole study.

## Results

The analysis of demongraphic data showed no significant difference (*p* > 0.05) in age between all three groups (Group A = 25.9 ± 2.02 years, Group B = 25.6 ± 2.06 years, Group C = 25.7 ± 2.07 years). Of the 20 participants in each group, there were 6 females in Group A and 5 females in each Groups B and C. The PRBB cognitive score (Group A = 79.2 ± 7.98, Group B = 79.05 ± 7.14, Group C = 82.22 ± 10.6) and total time taken to complete the cognitive task (Group A = 113.66 ± 12.84 s, Group B = 114.97 ± 8.67 s, Group C = 117.19 ± 11.69 s) were similar between group (*p* > 0.05). Details are shown in the Supplementary material (Table S-1). Twelve participants in Groups A and B reached the last round (Round 5 with 8 digits for memorizing) in PRBB state, while this number raised to 18 in Group A and reduced to 7 in Group B in POBB state. However, in Group C, ten and eleven participants reached the last round in PRBB state and POBB state, respectively.

We performed an interim analysis on first ten participants per group to calculate the efect size and estimate a reuqired number of participants per group. Statistical significance was set to *p* < 0.025 rather than *p* < 0.05 to avoid false positive, due to multiple comparison, i.e., the fact that date from first ten participatns were used twice, for the interim and for the final analysis. Based on a statistical analysis which was applied on first ten participants in each group, a large effect size (*d* = 0.95 for score and *d* = 0.926 for total time) and a statistical power (97.6% in score and 79.6% in time) was obtained in Group A, whilst a medium and a small effect size were observed for Groups B and C, respectively. The statistical power for both groups was less than 30%. Based on the statistical power analysis and the effect size of Group A, a sample size of 19 participants per group (total 57) was required for conducting this study when *p* < 0.025.

### Behavioral results

**Fig. 2:**
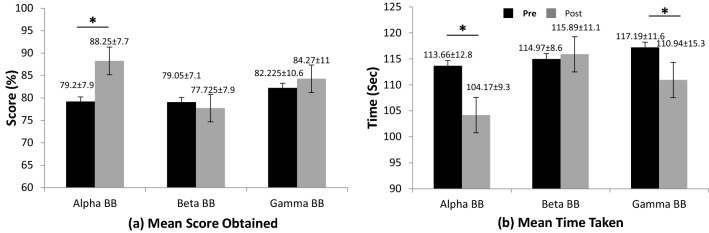
Comparison of participant’s performance for digit span test in pre (black bars) and post (gray bars) BB states for all three groups (alpha, beta, and gamma BB). **a** Mean score obtained by participants, and **b** mean time taken by participants to complete task. The significance level is set at *p* < 0.05 and is shown with * between PRBB and POBB states

Figure 2 demonstrates results of a digit span test for PRBB (black bars) and POBB (gray bars) states for all three groups (A, B, and C). The subfigure (a) shows mean score obtained by participants across all rounds while subfigure (b) shows mean time taken by participants across all rounds to complete the task. The mean value and standard deviation for both cognitive scores and time taken to complete tasks are shown at the top of each bar in Fig. 2. Also, the significant change in score and time in POBB state as compared to PRBB state are marked by an asterisk (*) at the top of each bar. For alpha BB in Group A, a mean score significantly increased (*p* = 0.0000235, *t*-value = 5.23) with a large effect size and statistical power (*d* = 1.15, SP = 0.991), while the time taken to complete the task significantly decreased (*p* = 0.0000157, *t*-value = − 5.41) with large effect size and the statistical power (*d* = 0.846, SP = 0.944) in POBB state as compared to PRBB state. Whereas with gamma BB, in Group C, only the time taken to complete the task showed a significant reduction (*p* = 0.014, *t*-value = − 2.37) with a medium effect size and statistical power (*d* = 0.46, SP = 0.545). No significant changes were found for beta BB in Group B.

Comparing behavioral performances between PRBB and POBB states for the fifth round (most complex round; required memorizing eight digits), a significant increase in cognitive scores (*p* = 0.0002) and a reduction in reaction time (*p* = 0.008) was noticed in Group A only.

**Fig. 3:**
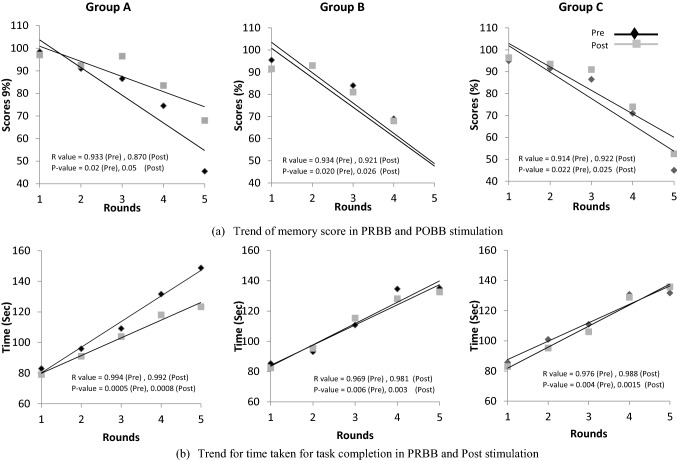
Regression line showing trend of memory score (subfigure ‘**a**’) and time taken to complete task (subfigure ‘**b**’) over five levels of digit span test in Pre (black line) and Post (gray line) states for three groups (Group A: alpha, Group B: beta, and Group C: gamma BB)

Figure 3a shows a regression line for memory scores obtained in each round for PRBB and POBB states for all three groups. In the first round, participants were asked to memorize four digits while in the last round (i.e., round 5) participants were asked to memorize eight digits. The slope of the score is significantly negative in both PRBB and POBB states in all three groups. The values of ‘*r*’ and ‘*p*’ are shown on Fig. 3a. However, the slope is more negative in the PRBB state as compared to the POBB state. The score difference (Post–Pre) is positive in later sessions 4–5 (participants can memorize more digits following stimulation). This means that BB training enables participants to improve their performance of complex tasks.

Figure 3b shows a regression line for response time obtained in each round for PRBB and POBB for alpha, beta, and gamma BB stimulation. The slope is significantly positive in both PRBB and POBB states, being larger in PRBB. The values of ‘*r*’ and ‘*p*’ are shown on Fig. 3b. Also, the time difference (Post–Pre) is negative in later sessions as compared to early sessions. This indicates enhanced participants’ ability to perform a complex task.

### EEG power

**Fig. 4:**
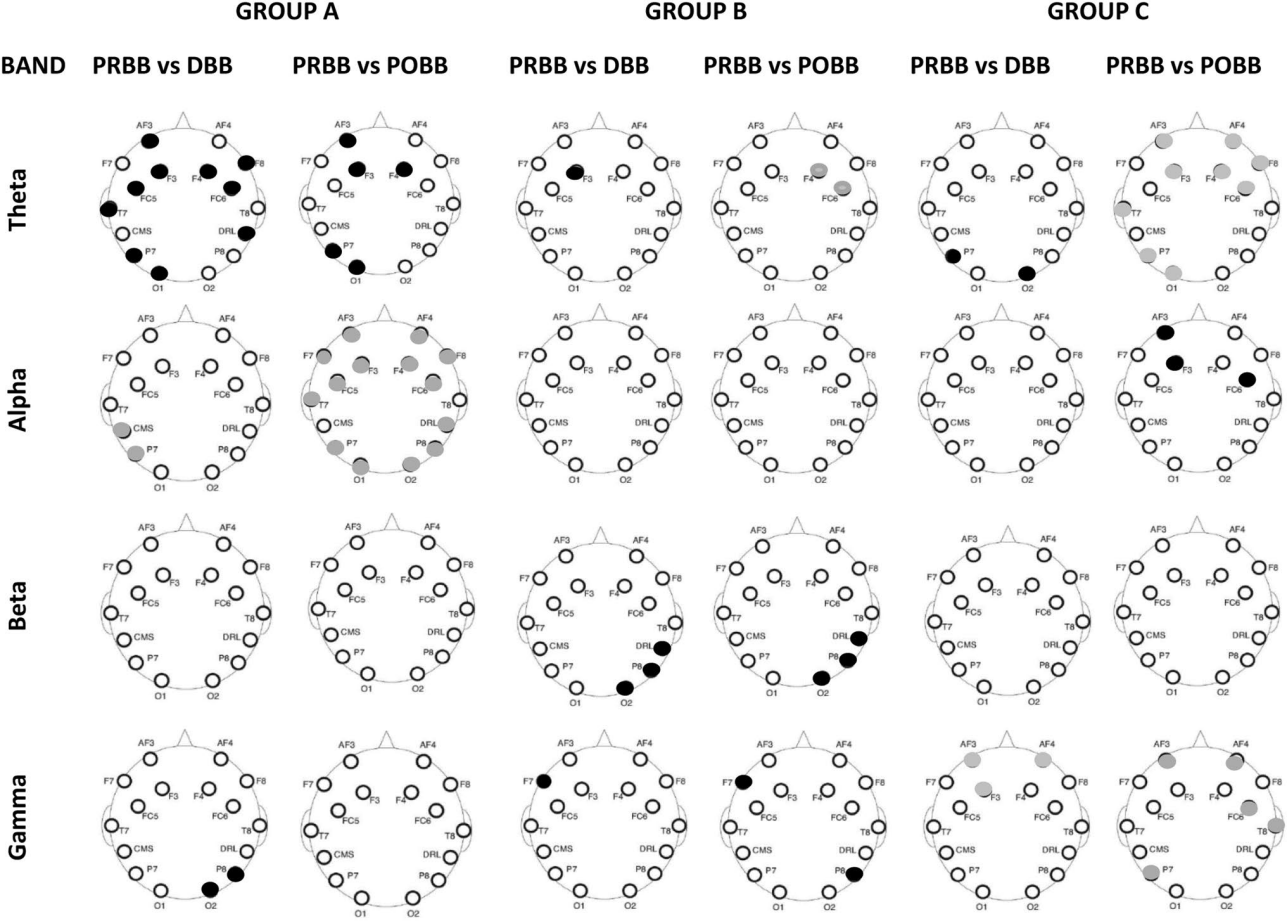
Statistically significant difference in EEG power between two states (PRBB vs DBB, and PRBB vs POBB) for three groups (A, B, and C) in the theta, alpha, beta, and gamma frequency bands. Rows represent frequency bands (first row: theta, second row: alpha, third row: beta, fourth row: gamma). First three columns represent statistically significant difference between PRBB and DBB states and between PRBB and POBB state for Groups A, B, and C, respectively. Black color represent decrease significant power while gray color increase significant power in DBB and POBB states as compared to PRBB state

Figure 4 shows scalp maps with statistically significant increase (black dots) or decrease (gray dots) of EEG power in different frequency bands as compared to PRBB, for all three groups (*p* < 0.05, after a correction for multiple comparisons, performed separately for each group).

In Group A, the prominent features are a widespread increase of the alpha power in both DBB and POBB states and theta band decrease of power in DBB POBB state.

In Group B, the main changes in power occur in the beta band in the right parietal region, which decreases in both DBB and POBB states that could be related to BB frequency of stimulation in the beta range at 14 Hz. In contrast to the other two groups, in Group B, changes in EEG power are very similar in DBB and POBB.

In Group C, that had BB stimulus at 30 Hz, that corresponds to the lower gamma range, one can observe an increase in the frontal gamma band power during the DBB state and an increase in both theta and gamma power in the bilateral frontal and left parietal cortex during POBB state.

In summary, Groups A and C that have improvement in either cognitive score or in reaction time showed an increase in DBB power in the frequency range corresponding to BB frequency. Both groups also showed changes in theta band power in POBB but in different directions. In Group C which shows an increase in response time, a simultaneous increase of theta and gamma band power can be noticed.

### Imaginary coherence

**Fig. 5:**
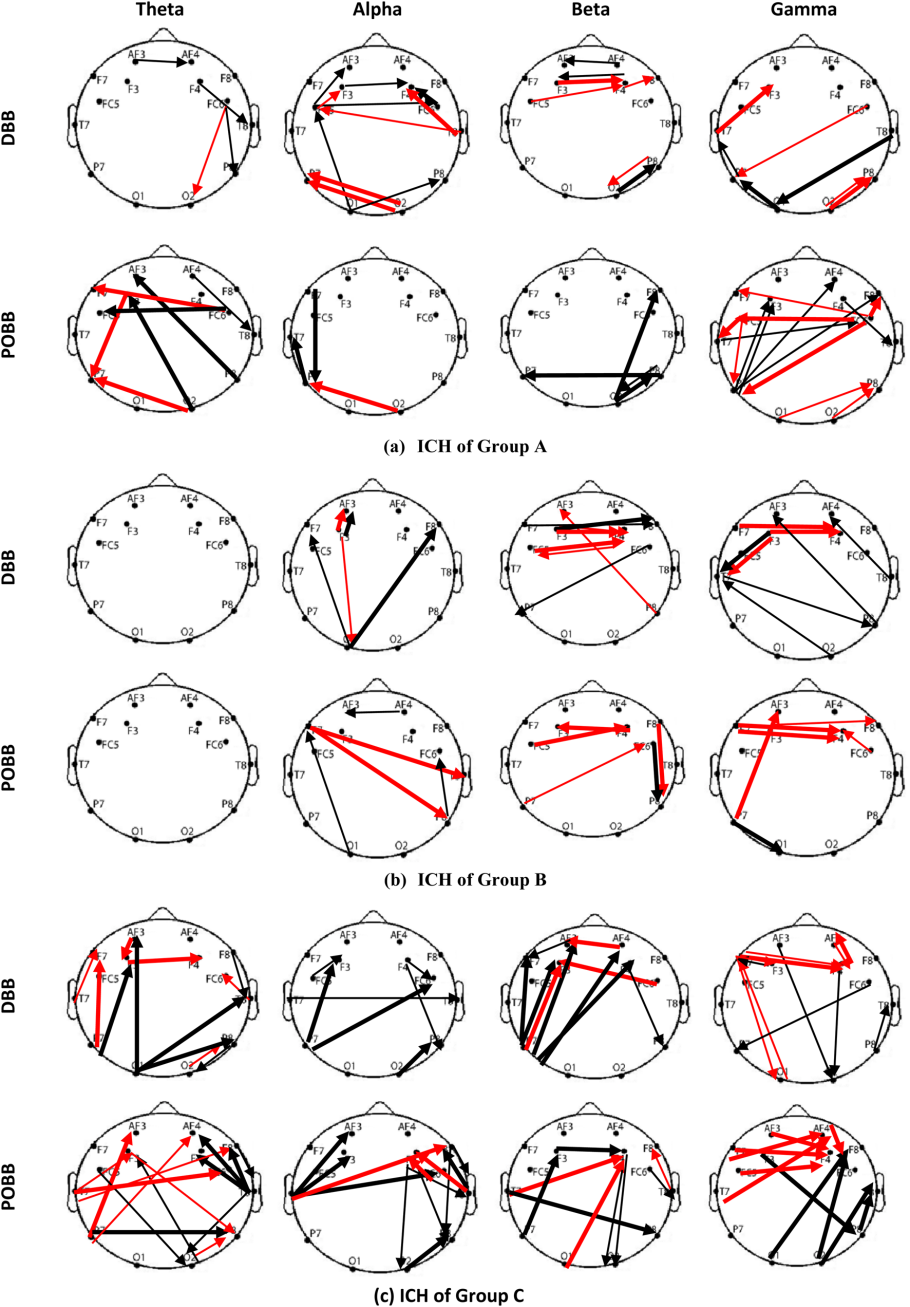
Imaginary coherence (ICH) between DBB and PRBB and between PRBB and POBB states in the theta, alpha, beta, and gamma bands for three groups (‘**a**”; Group A, ‘**b**’; Group B, and ‘**c**’; Group C). The thick/thin line means increase/decrease strength in POBB and DBB state as compared to PRBB state. The black arrow represents the same direction of connection or information flow from one region to the other region and red arrow represent the change in the direction of connectivity or information flow in POBB and DBB as compared to PRBB

Figure 5 presents ICH that shows statistically significant differences between DBB & PRBB, and PRBB & POBB for Group A, B, and C, respectively. ICH is presented in the theta, alpha, beta, and gamma bands for all three groups. For each comparison we show ICH with two different line thickness: thicker for coherence values which got stronger as compared to PRBB and thinner for coherence values which become weaker as compared to PRBB.

#### PRBB vs DBB alpha stimulation

Across all frequency bands most ICH become weaker in DBB, apart from localized connections in the occipital regions, across several frequency bands (8–40). The largest number of significant changes in ICH can be noticed in the alpha band that is a frequency in which BB was delivered. Most ICH in the alpha band become weaker in the active state (DBB) as compared to the baseline. A large number of reduced, frontally localized interhemispheric ICH in DBB was accompanied with increased interhemispheric ICH in the occipital area.

#### PRBB vs POBB alpha stimulation

POBB ICH significantly increased in the theta band and decreased in the gamma band.

Two networks were affected in both the theta and gamma band: a long-range bilateral parieto-frontal and localized, interhemispheric frontal network.

Very few differences between PRBB and POBB state were found in the alpha and beta band.

It is interesting to note that while PRBB vs POBB are characterized with a widespread significant increase of alpha power, this increase is not reflected in changes in coherence. On the other hand, theta band power decreased in POBB state while coherence increased.

#### PRBB vs DBB beta stimulation

DBB localized frontal interhemispheric ICH significantly increased (short-range) in the alpha, beta and gamma bands while long-range connectivity decreased in alpha, beta, and gamma bands. No significant change was noticed in the theta band connectivity.

#### PRBB vs POBB Beta stimulation

POBB ICH significantly increased in alpha, beta, and gamma frequency bands. In the alpha band, a long-range interhemispheric ICH increased between the left frontal and the right centro-parietal areas. In the beta band, increased coherence could be noticed within the frontal region and between frontal and parietal regions over the right hemisphere. In the gamma band, increase in ICH was localized to the frontal cortex.

#### PRBB vs DBB gamma stimulation

DBB ICH significantly increased between the frontal and posterior regions (long-range differences originated from the posterior region) in the theta and beta bands. The increased connectivity in the theta band could also be noticed within the frontal and within posterior region. In both alpha and beta bands, there was an increased ICH from the left parietal to both left and right frontal areas and decreased ICH from the right frontal to the right parietal cortex. In the gamma band, local interhemispheric frontal ICH increased while long-range ICH decreased in a direction from frontal towards the occipital regions.

#### PRBB vs POBB gamma stimulation

The connectivity strength in theta, alpha, and beta bands shows complex patterns of simultaneously increasing and decreasing ICH while connectivity in the gamma band only increased. The increase in strength could be observed between the right temporal and frontal and between the left and right parietal regions in the theta band, between the frontal and parietal and the frontal and temporal, and within the posterior region in the alpha band, among the temporal, frontal, and posterior regions in the beta band, within the frontal and between the frontal and posterior regions in the gamma band. A decrease in strength is observed in theta, alpha, and beta bands between the frontal and posterior regions.

Comparing ICH between DBB and POBB in Groups A, B, and C one can notice that there is no clear relationship between the frequency of BB and changes in ICH in the corresponding frequency band. BB at 14 Hz in Group B results in the smallest changes based on the number of connections that show significant differences between DBB and PRBB.

#### ICH sources and sinks

The number of sources and sinks was calculated in DBB and POBB states in the theta, alpha, beta, and gamma bands for three regions of the brain (anterior, temporal, and posterior) for Groups A, B, and C. The N way ANOVA analysis (*p* = 0.05) was calculated for sinks and sources separately. The number of sinks and sources were independent variables while states, frequency bands (theta 4–8 Hz, alpha 8–13 Hz, beta 14–30 Hz, gamma 30–49 Hz), brain regions (anterior, occipito-posterior and temporal) and groups were four factors.

Detailed results are presented in Supplementary material (Table S-2).

##### Sources

Following Holm–Bonferroni correction for multiple comparison, there was a statistically significant difference between regions (*p* = 3.81E−13), states (*p* = 4.8E−13), frequency bands (*p* = 0.003855) and groups (*p* = 7.23E−66). There was also a statistically significant difference for the interaction between regions and states (*p* = 1.31E−05), regions and frequency bands (*p* = 2.19E−10), regions and groups (*p* = 0.000305), states and groups (*p* = 2.94E−09) and frequency bands and groups (*p* = 4.86E−24). The only non-significant relation was between the states and frequency bands (*p* = 0.034371).

A pair-wise comparison revealed a significant difference in theta, beta, and gamma bands between all three groups in DBB and a significant difference between sources in all three frequency bands between each pair of groups in POBB. There are no significant differences between groups in the alpha band for DBB.

There was also a significant difference between sources in theta, beta, and gamma bands between each pair of three groups (A vs B, B vs C, A vs C) in DBB and a significant difference between sources in all four frequency bands between each pair of groups in POBB. All pair-wise results are based on Holm–Bonferroni correction for multiple comparisons.

##### Sinks

Following Holm–Bonferroni corrections for multiple comparison there was a statistically significant difference between regions (*p* = 1.95E−45), states (*p* = 1.74E−13), frequency bands (*p* = 5.93E−18) and groups (*p* = 3.45E−62). There was also a statistically significant difference for the interaction between regions and frequency bands (*p* = 3.83E−11), regions and groups (*p* = 1.29E−27), states and frequency bands (*p* = 7.03E−07), states and groups (*p* = 3.24E−15), and frequency bands and groups (*p* = 6.46E−18). The only non-significant relation was between the regions and states (*p* = 0.217012).

A pair-wise comparison revealed a significant difference between each pair of groups in all frequency bands in DBB. The only exception was a difference between Groups B and C in the alpha band which did not show statistically significant difference.

There was also a significant difference between each pair of groups in all frequency bands in DBB. The only exception is difference between Groups A and C in the gamma band which did not show statistically significant difference. All pair-wise results are based on Holm–Bonferroni correction for multiple comparisons.

## Discussion

This study compares the effectiveness of three different frequencies of BB stimulation on a short term memory (working memory) and analyses related neurological changes with the aim to establish a putative mechanism of BB. Behavioral changes were measured in terms of memory recall and reaction times while neurological changes were analyzed in terms of EEG power and ICH in different frequency bands, in DBB and POBB state as compared to PRBB state. The study shows that BB in alpha and higher beta/lower gamma range result in behavioral changes, as opposed to BB in beta band. Our results are in accordance with previous studies showing that alpha BB improves memory in healthy people and people with Alzheimer's and Parkinson's disease (Calomeni et al. 2017; McMurray 2006). However, while most studies were aiming to establish whether or not the frequency of BB can induce changes in the corresponding frequency band, we look further to explore the relationship between the specific BB frequency and related changes in cortical networks underpinning memory consolidation.

The results indicate that two types of BB stimulation which improved performance are both associated with the increase in theta ICH. The increased fronto-parietal connectivity in the theta band found in Groups A and C was also previously reported in 1 Hz, 10 Hz, and 20 Hz BB stimulations (Beauchene et al. 2016, 2017; Gao et al. 2014). In Beauchene et al., only 20 BB that resulted in behavioral changes was accompanied with increased fronto-parietal connectivity (measured by a phase locking value) and both alpha and beta BB resulted in increased theta band power. Gao et al. (2014) analyzed a minute-by minute response to 5 min BB and also found increase in phase locking values between the frontal and occipital/temporal regions after several minutes of alpha and beta BB. It is believed that a coupling between the pre-frontal and temporo-parietal brain areas in the theta band is fundamental for encoding and retrieval of working memory task (Sauseng et al. 2009).

Improved visual working memory was important for a digit span task performed in this study. It is believed that visual working memory is under control of the pre-frontal cortex (Voytek and Knight 2010). Increased ICH interconnectivity within pre-frontal regions in DBB and POBB, in beta and gamma range helped regulating the flow of information that contributed to improved performance in a digit span task.

Alpha activity is positively associated with the quality of performance while processing higher cognitive functions such as working memory. Previous studies also reported association of reduced theta activity and enhanced memory score (Klimesch 1999). In line with this, we found an increased cognitive score followed by enhanced alpha and reduced theta activity with alpha BB (Group A). Alpha power increase may be related to alpha entrainment with 10 Hz BB.

Gamma band BB showed the largest number of significant changes (either increase or decrease) in ICH in both DBB and POBB states that may also be related to behavioral changes. For example, Group B that showed no behavioral changes also had the smallest number of statistically significant changes in ICH. Although a cross-frequency coupling was not investigated in this study, the concomitant increase of theta and gamma ICH in POBB during alpha and gamma BB is indicative of such processes (Lisman and Jensen 2013).

Furthermore, the improved reaction time in Groups A and C is in line with the results of previous studies showing that alpha and gamma BB improved a reaction time (Colzato et al. 2018; Shekar et al. 2018).

There was no improvement of cognitive task in Group B despite an increase in theta and gamma power POBB at few electrodes, which is in line with some previous studies (Goodin et al. 2012; Vernon et al. 2014) but contradict others (Beauchene et al. 2016; Kennerly 1994; Kraus and Porubanová 2015). This suggests that the benefits may be frequency specific. Contradictory findings may be due to differences in study design which include different procedures for providing BB stimulation (continuous, even-related, combined with noise, music), variations in the number and length of sessions, the frequency of stimulation, and the selection of frequency range for stimulation (Wahbeh et al. 2007).

For example, Kennerly (1994) reported improvement of memory tasks with beta BB but they combined beta BB with music and did not provide information about the carrier frequency or total duration of the session. A 5 min of BB on 5 Hz, 10 Hz and 15 Hz with a carrier frequency of 240 Hz was tested in experiments testing visuospatial working memory (Beauchene et al. 2016) and verbal working memory (Beauchene et al. 2017). Both studies reported an improvement in performance for beta BB only. However, both studies tested all three frequency BB on the same group, potentially having a carry over or cumulative effect on 15 Hz BB that was presented last. A meta-analysis found that every 1 min of BB increases the effect size for 0.01 (Garcia-Argibay et al. 2019). However, a consistent finding between Beauchene et al. 2016, 2017 and current study is that BB that results in significant behavioral improvement also results in increased frontal parieto/temporal coupling.

To better understand the mechanism of BB stimulation and modification in brain networks following memory enhancement with BB stimulation, it is vital to study the brain regions involved in processing working memory and auditory stimulation (Smith and Jonides 1999; Vogel et al. 2005). In this regard, imaginary coherence analysis performed in this study indicates that BB stimulation does not only induce changes in the numbers and strength of connections but also in the directions of information flow between different brain networks.

The frontal cortex itself and its interaction with the parietal cortex is considered to be strongly associated with working memory performance and is also responsible for planning the appropriate behavioral responses to external and internal stimuli (Curtis and D’Esposito 2003; Postle 2006). These functions are weakened in DBB state as shown by weakening the frontal theta ICH in DBB state, accompanied by increased ICH of the frontal beta and gamma band. However, in spite of this, in the theta and gamma bands, large-scale (fronto-parietal) connectivity changes were noticed in POBB state. Changes in the large-scale/long-range cortical network of oscillatory synchronization in the theta and gamma bands reported in this study play an important role in various cognitive functions such as selective attention (Gregoriou et al. 2009; Saalmann et al. 2007; Siegel et al. 2008), decision making (Pesaran et al. 2008), emotion (Costa et al. 2006), and working memory (Fell and Axmacher 2011; Palva et al. 2010; Sauseng et al. 2008).

Theta and gamma frequency oscillations occur in the same brain regions and interact with each other, a process called cross-frequency coupling (Lisman and Jensen 2013). Their coupling is important for long term memory consolidation (Friese et al. 2013), but that hypothesis was not tested in the current study. However, it is interesting to note that gamma connectivity is decreased and theta connectivity is increased in this study for alpha BB. This decrease in gamma connectivity was also reported in a study conducted by Burke et al. (2013) and it represents synchronous multi-unit neural gamma activity (Solomon et al. 2017). The gamma band also plays an important role in the attention and maintenance of relevant memory (Sauseng et al. 2009). This concomitant decrease of ICH in the gamma band is surprising, as such decoupling of brain regions in healthy people typically occurs during REM sleep (Castro-Zaballa et al. 2019). Weakened ICH from occipital towards parietal and central regions bilaterally may indicate a disruption of a default mode network as a result of BB. Moreover, different responses in the gamma band responses with alpha (Group A) and gamma BB (Group C) stimulations may demonstrate the segregation of gamma activity into oscillations and asynchronous processes.

BB stimulation within theta frequency range was not included in this study, though theta band activity is related to the performance of the working memory and also showed the largest changes in this study. Published literature reported varied effects of theta BB stimulation, with most studies reported no effect (Garcia-Argibay et al. 2019).

A limitation of this study is a relatively short duration of a single training without a separate control group, and a small number of EEG electrodes preventing in depth analysis of activity of deeper cortical structures. A lack of improvement in cognitive performance accompanied with the lack of neurological effect in beta BB and a consistent change in theta ICH in alpha and gamma BB indicate that the effect of BB was frequency specific and not the effect of some general increased attention processes.

## Conclusions

The results of this study indicate the neurological mechanism through which BB may affect memory recall tasks is related to theta band power and ICH. A single training session of 15 min might be enough to induce enhanced memory. Due to a simple experimental setup BB could easily be applied in clinical settings. Further studies with larger numbers of electrodes and experimental sessions would be required to establish its long term effectiveness.

## Supplementary Information

Below is the link to the electronic supplementary material.

Supplementary file1 (DOCX 15 kb)

Supplementary file2 (DOCX 17 kb)
