# Supplementary material for: Understanding the neurological mechanism involved in enhanced memory recall task following binaural beat: a pilot study
Source: Exp Brain Res. 2021 Jul 7;239(9):2741–54. doi: 10.1007/s00221-021-06132-6 (PMC8448692; doi:10.1007/s00221-021-06132-6)
Supplement: Supplementary file 1 — Supplementary file1 (DOCX 15 kb) [file 221_2021_6132_MOESM1_ESM.docx]

**Table S-1:** Demographic Information (Age, Gender, Pre data)

| **S.No** | **Alpha BB** | | | | **Beta BB** | | | | **Gamma BB** | | | |
| --- | --- | --- | --- | --- | --- | --- | --- | --- | --- | --- | --- | --- |
|  | **Age** | **Gender** | **Pre Score** | **Pre Time Taken** | **Age** | **Gender** | **Pre Score** | **Pre Time Taken** | **Age** | **Gender** | **Pre Score** | **Pre Time Taken** |
| **1** | 25 | Male | 78 | 110.8 | 26 | Male | 84 | 115.4 | 26 | Male | 74 | 109.6 |
| **2** | 26 | Male | 92 | 109.2 | 24 | Male | 84 | 120.6 | 26 | Male | 60 | 118.6 |
| **3** | 24 | Male | 72 | 104.8 | 30 | Male | 67.5 | 106.25 | 27 | Female | 76 | 141.2 |
| **4** | 24 | Female | 74 | 107 | 22 | Male | 94 | 110.2 | 25 | Female | 86 | 107.8 |
| **5** | 27 | Male | 72 | 141.8 | 27 | Male | 76 | 126.4 | 24 | Male | 74 | 109.6 |
| **6** | 25 | Female | 82 | 109.4 | 29 | Male | 80 | 116 | 27 | Male | 94 | 106.2 |
| **7** | 23 | Female | 88 | 112.6 | 25 | Female | 72 | 122 | 30 | Male | 90 | 118.4 |
| **8** | 25 | Male | 64 | 138.2 | 25 | Female | 85 | 119.6 | 30 | Male | 67.5 | 116.75 |
| **9** | 27 | Male | 92 | 114.8 | 23 | Male | 78 | 129.2 | 24 | Male | 74 | 134.4 |
| **10** | 24 | Male | 76 | 113.6 | 25 | Male | 80 | 122.8 | 25 | Male | 80 | 120.4 |
| **11** | 26 | Male | 76 | 118.8 | 26 | Male | 88 | 131 | 27 | Male | 80 | 135 |
| **12** | 30 | Male | 80 | 105 | 27 | Male | 80 | 106.8 | 28 | Male | 90 | 123.4 |
| **13** | 31 | Male | 90 | 94.8 | 24 | Male | 74 | 108.8 | 22 | Male | 70 | 134.3 |
| **14** | 27 | Female | 84 | 99.4 | 24 | Female | 86 | 100.8 | 24 | Female | 92 | 101.2 |
| **15** | 28 | Male | 72 | 101.4 | 26 | Male | 80 | 106.5 | 25 | Female | 86 | 106.4 |
| **16** | 25 | Female | 68 | 110.2 | 28 | Female | 62.5 | 107.25 | 25 | Female | 85 | 120.4 |
| **17** | 26 | Female | 82 | 103.8 | 25 | Female | 80 | 104.8 | 26 | Male | 100 | 108.8 |
| **18** | 25 | Male | 76 | 125.8 | 24 | Male | 74 | 117.4 | 24 | Male | 100 | 104.6 |
| **19** | 26 | Male | 88 | 115.8 | 28 | Male | 80 | 117.4 | 23 | Male | 80 | 120.4 |
| **20** | 24 | Male | 78 | 136 | 24 | Male | 76 | 110.2 | 26 | Male | 86 | 106.4 |
