# Supplementary material for: Understanding the neurological mechanism involved in enhanced memory recall task following binaural beat: a pilot study
Source: Exp Brain Res. 2021 Jul 7;239(9):2741–54. doi: 10.1007/s00221-021-06132-6 (PMC8448692; doi:10.1007/s00221-021-06132-6)
Supplement: Supplementary file 2 — Supplementary file2 (DOCX 17 kb) [file 221_2021_6132_MOESM2_ESM.docx]

**Table S-2 Four-way ANOVA analysis on numbers of sources and sinks**

| **Factors** | **Source** | | **Sink** | |
| --- | --- | --- | --- | --- |
|  | **F** | **p Value** | **F** | **p Value** |
| **Regions (Anterior, Temporal, Posterior)** | 30.51 | 3.81E-13 | 113.7 | 1.95E-45 |
| **State (DBB,POBB)** | 55.28 | 4.8E-13 | 56.21 | 1.74E-13 |
| **Frequency Bands (Theta, Alpha, Beta, Gamma)** | 4.56 | 0.003855 | 29.36 | 5.93E-18 |
| **Groups (A,B,C)** | 172.21 | 7.23E-66 | 161.51 | 3.45E-62 |
| **Regions*States** | 11.7 | 1.31E-05 | 1.53 | 0.217012 |
| **Regions*Frequency Bands** | 9.89 | 2.19E-10 | 10.37 | 3.83E-11 |
| **Regions*Groups** | 5.43 | 0.000305 | 35.5 | 1.29E-27 |
| **States*Frequency bands** | 2.89 | 0.034371 | 10.67 | 7.03E-07 |
| **States*Groups** | 20.48 | 2.94E-09 | 34.8 | 3.24E-15 |
| **Frequency Bands*Groups** | 22.01 | 4.86E-24 | 16.36 | 6.46E-18 |

**Table S-3:** Pairwise comparison for total numbers of source and sink between frequency bands and between groups. Significant values (P<0.05) represented by bold numbers

| **Bands** |  | **DBB** | | | | | | **POBB** | | | | | |
| --- | --- | --- | --- | --- | --- | --- | --- | --- | --- | --- | --- | --- | --- |
|  |  | **Group A and B** | | **Group A and C** | | **Group B and C** | | **Group A and B** | | **Group A and C** | | **Group B and C** | |
|  |  | **t-Value** | **p-Value** | **t-Value** | **p-Value** | **t-Value** | **p-Value** | **t-Value** | **p-Value** | **t-Value** | **p-Value** | **t-Value** | **p-Value** |
| **Theta** | **Source** | 4.729 | **1.4E-05** | -3.42 | **0.0007** | -9.807 | **1E-06** | 8.208 | **1E-06** | -5.19 | **3.2E-06** | -11.47 | **1E-06** |
|  | **Sink** | 3.370 | 0.0009 | -3.06 | 0.003 | -4.703 | 1.6E-05 | 9.059 | 1-06 | -5.08 | 4.8E-06 | -7.341 | 1E-06 |
| **Alpha** | **Source** | 0 | 0.5 | 0.000 | 0.5 | 0 | 0.5 | -3.679 | **0.0003** | -6.16 | **1E-06** | -4.386 | **4.2E-05** |
|  | **Sink** | -2.10 | 0.02 | -1.88 | 0.03 | 0 | 0.5 | -5.703 | 1-06 | -8.96 | 1E-06 | -8.195 | 1E-06 |
| **Beta** | **Source** | -5.38 | **1.8E-06** | -5.83 | **1E-06** | -4.300 | **5.2E-05** | -3.679 | **0.0004** | -9.81 | **1E-06** | -7.379 | **1E-06** |
|  | **Sink** | -6.12 | 1E-06 | -5.85 | 1E-06 | -4.280 | 6.3E-05 | -3.497 | 0.0008 | -5.79 | 1E-06 | -3.432 | 0.0008 |
| **Gamma** | **Source** | -3.85 | **0.0003** | -5.02 | **5.5E-06** | -2.473 | **0.008** | 10.038 | **1E-06** | 3.268 | 0.001 | -7.881 | **1E-06** |
|  | **Sink** | -2.56 | 0.008 | -2.60 | 0.007 | 0 | 0.5 | 11.278 | 1E-06 | 0 | 0.5 | -10.51 | 1E-06 |
